# Perceptions of Aging in the Hispanic Community Members in South Central United States: A Descriptive and Exploratory Analysis

**DOI:** 10.1155/jare/4792626

**Published:** 2026-04-16

**Authors:** Ethan Bradford, Basilia Oseguera, Mackenzie Reed, Shakshi Sharma, Lou Ann Eads, Jeanne Wei, Gohar Azhar

**Affiliations:** ^1^ Department of Geriatrics, University of Arkansas for Medical Sciences, Little Rock, Arkansas, USA, uams.edu; ^2^ Department of Psychiatry, University of Arkansas for Medical Sciences, Little Rock, Arkansas, USA, uams.edu

## Abstract

The aim of this study is to investigate perceptions of aging among Hispanic adults. Fifty participants in Texas and Arkansas were asked to take a short questionnaire including multiple‐choice and Likert scale format questions. Individuals 18 and older who identified as Hispanic were included in the study. Counts and frequencies for each response were obtained. Furthermore, an exploratory comparison of responses between younger and middle‐aged/older respondents was conducted. According to the results of the study, the Hispanic adults surveyed were found to have a generally positive view of aging, most frequently associating older age with the words “Wisdom” (68%) and “Experience” (12%). However, respondents still had anxieties about aging, with many answering that they feared death (41.3%), loss of independence (41.7%), and financial issues (31.3%) often or always. Additionally, the majority of those surveyed indicated that they would prefer to live with their spouse only in old age, which may reflect a generational difference in attitudes toward living arrangements. Keeping these results in mind will be essential for healthcare providers as they aim to develop culturally competent educational programs to relieve caregiver burden and support healthy aging for the rapidly growing older Hispanic population in the United States.

## 1. Introduction

Geriatrics is an expanding field in healthcare. Life expectancy has nearly doubled in the 20th century [1]. By 2030, one in six people will be aged 60 years or older, and in 2034, people aged 65 and older will outnumber those under the age of 18 [2, 3].

As the global population ages, understanding and addressing ageism will become increasingly important. Ageism refers to the stereotyping, prejudice, and discrimination directed at an individual because of their age [4]. Ageism is a widespread issue: A recent study revealed that over 90% of adults 50 to 80 years old reported experiencing at least one form of ageism daily [5–7]. In addition to experiencing external forms of ageism, older adults can harbor internal beliefs about aging. Stereotype embodiment theory suggests that when external beliefs are internalized by an individual, their manifestation in that person can influence their health. This process can occur top‐down or over the course of one’s lifespan.

Numerous studies suggest that possessing a negative self‐stereotype is associated with worsened cardiovascular health and may be associated with overall poor health outcomes and higher mortality rates [8–11]. On the other hand, it has been suggested that internalizing positive beliefs about aging may act as a protective factor against developing cognitive decline or dementia [12]. Several factors, including dignity, education, and living with others, correlated to having a more positive outlook on aging [13]. These findings highlight the importance of understanding how aging is perceived across different social and cultural contexts. Previous research has demonstrated meaningful differences in perceptions toward aging between younger and older adults [14]. However, while there have been multiple studies since the 1950s about opinions and attitudes about aging in general in the United States, previous reviews have failed to assess how this differs across cultures [15, 16]. Furthermore, there is a dearth of studies examining how perceptions of aging may differ across the life course between younger and older adults within the same cultural group.

In the United States, Hispanics account for 19% of all Americans and are the second largest racial or ethnic group. They are also one of the fastest‐growing groups in the United States, with the country’s Hispanic population growing 23% between 2010 and 2020 [17]. The Census Bureau estimates that the Hispanic population aged 65 and older will increase fivefold by 2050 [18]. When caring for a patient and their family, it is critical to understand their culture, and there is evidence that supports that improving cultural competency may improve patient health outcomes [19]. Specifically, enhanced cultural competency has been theorized to increase patient satisfaction and trust and improve adherence to treatment [20].

Three aspects of Hispanic culture—familismo, personalismo, and respeto—are key to understanding family dynamics, especially as it pertains to older adults in their culture. Familismo emphasizes the importance of family cohesion and unity; personalismo refers to the value placed on interpersonal relations within the family; and respeto underlines the importance of maintaining respect within this dynamic, especially as it concerns reverence and respect for elders [21]. These values possibly function as sources of strength by promoting social support and involvement of older adults in community life. Hispanic communities tend to have extended family networks, and caregiving for family members is very common, with over a third of Hispanic households in the United States reporting caring for at least one elderly family member [22]. As such, Hispanics aged 65 and older are much more likely to live with other relatives than live with nonfamily members or live alone than non‐Hispanic whites [23].

Importantly, these values are not experienced uniformly across Hispanic communities, and additional factors such as country of origin, immigrant status, and acculturation status may significantly influence perceptions of aging and health outcomes [24]. Although these factors were not measured in the present study, acknowledging this heterogeneity is important for interpreting findings and guiding future research. Additionally, for Hispanic communities in the United States, health outcomes and perceptions toward aging are shaped not only by cultural values but also by systemic factors such as socioeconomic inequities, immigration‐related barriers, and access to healthcare [25].

When compared to non‐Hispanic Whites in the United States, Hispanics typically have higher rates of obesity, diabetes, liver disease, dementia, and depression [26]. Among older Hispanic adults, a higher proportion report having at least one of seven chronic health conditions: asthma, cancer, heart disease, diabetes, hypertension, obesity, or anxiety/depression [27, 28]. Even with these higher rates of chronic health conditions, Hispanics have lower overall mortality rates compared to non‐Hispanic Whites; this is known as the “Hispanic Paradox.” The “Hispanic Paradox” refers to the widespread evidence of lower socioeconomic status and high rates of chronic health conditions but low all‐cause and infant mortality rates [29, 30]. Understanding perceptions of aging is particularly important in this context, as positive beliefs and cultural strengths may support well‐being despite systemic barriers [31].

Limited cultural awareness, health literacy, and Hispanic healthcare providers pose the largest barriers to healthcare access [32, 33]. In addition, late middle‐aged and older Hispanic adults are more likely to perceive medical discrimination because of race and age simultaneously, highlighting the importance of examining the intersection of these two factors [34].

While much research on aging has centered on general attitudes and perspectives, less attention has been given to how aging is perceived within the Hispanic community, especially among younger Hispanic adults. While a study from the AARP published in 2019 did reveal that young Hispanic adults aged 18–64 were more positive about turning 50 than non‐Hispanics, research examining how younger Hispanic adults perceive aging and older adults more broadly is limited [35]. This is a significant issue, as perceptions of aging can have vast implications for long‐term health. Additionally, there is a gap in knowledge of the perceptions of aging between younger and middle‐aged/older Hispanic adults. By examining how this population views aging and older adults, this study aims to identify culturally rooted strengths, positive or negative beliefs, and areas where intervention may be able to enhance aging‐related well‐being.

## 2. Materials and Methods

### 2.1. Study Design

This study utilized a cross‐sectional, convenience sample design. Recruited participants were asked to complete a questionnaire covering their opinions regarding aging. Survey questions presented were in a single‐selection multiple‐choice or Likert scale format and were administered in English. Survey questions were newly developed by researchers at the University of Arkansas for Medical Sciences (UAMS). The survey was not formally validated for cultural appropriateness. Items were intentionally presented in plain language to reduce response burden and were reviewed by clinicians and social workers prior to data collection. This study was approved by the Institutional Review Board at UAMS (IRB#:217886). The complete survey is available upon request and may be permitted for use in further research.

### 2.2. Participants

Fifty Hispanic adults in Central Arkansas and Dallas, Texas, were invited to take the survey. All participants were required to be over the age of 18 and identify as Hispanic. There were no other eligibility criteria. Participants were recruited in person at community events, classrooms, college functions, or healthcare functions and facilities. Therefore, many of the participants may have been students. Recruitment occurred primarily in community venues serving Mexican American and other Central American populations. However, specific Hispanic subgroup information (e.g., country of origin and acculturation) was not collected. Participants were informed about the study and provided consent. All study subject material was kept anonymous and confidential.

### 2.3. Statistical Analysis

As all items were presented with categorical answers, summary statistics obtained include percentages and counts of responses to each question. To compare responses between young and middle‐aged/older Hispanic adults, participants were divided into groups below 40 years old and above 40 years old, and percentages and counts to each question stratified by age group were obtained. This cutoff allows sufficient sizes in each age group to be maintained while using a common breakpoint for the transition from young adulthood to middle‐aged. Answers to ordinal Likert scale items were recoded as positive integer values (e.g., 1, 2, 3, and 4), and Mann–Whitney *U* tests were conducted. Ordinal Cronbach’s alpha was obtained as a measure of scale reliability for the 17 Likert scale items.

This study is largely exploratory, and as many statistical tests are being performed, the Benjamini–Hochberg procedure was used to adjust critical values at a false discovery rate of 0.05 with 17 comparisons [36]. Critical values were calculated as (*i*/*m*) × *Q*, where *i* = ascending rank of the *p* value, *m* = # of comparisons, and *Q* = false discovery rate.

Statistical analysis was conducted in R Version 4.4.2, and data visualizations were created using the ggplot2 package [37, 38]. Due to the small number of observations, missing and invalid data were handled using pairwise deletion, and imputation methods were not considered.

## 3. Results

### 3.1. Participant Characteristics

Out of 50 participants, 64% were female and 36% were male. All participants were White and Hispanic or Latino. The age distribution of participants includes those less than 20 years old (22%), 20‐ to 29‐year‐olds (28%), 30‐ to 39‐year‐olds (20%), 40‐ to 49‐year‐olds (16%), 50‐ to 59‐year‐olds (8%), 60‐ to 69‐year‐olds (4%), and 70–79‐year‐olds (2%), indicating a sample with a young adult majority. Educational and household income backgrounds among participants were relatively diverse (Table 1). While specific information on country of origin was not gathered, surveys were gathered largely in Mexican American or other Central American communities in Dallas, Texas, and Central Arkansas.

**TABLE 1:** Participant demographics (*N* = 50).

	*N* (%)
*Gender*	
Female	32 (64)
Male	18 (36)

*Age group*	
< 20	11 (22)
20–29	14 (28)
30–39	10 (20)
40–49	8 (16)
50–59	4 (8)
60–69	2 (4)
70–79	1 (2)

*Household income*	
< $30,000	17 (34)
$30,000–45,001	4 (8)
$45,001–60,001	6 (12)
$60,000–100,000	8 (16)
> $100,000	4 (8)
Student	10 (20)

*Education*	
Less than high school	9 (18)
High school	21 (42)
Some college	7 (14)
College graduate	6 (12)
Postgraduate	3 (6)
Graduate degree	1 (2)

### 3.2. Descriptive Results for All Participants

Participants were asked to rank terms associated with the word “old” in order of importance. Overall, 68% of respondents ranked “Wisdom” as the most thought‐of term, followed by “Experience” (12%), “Respect” (8%), “Weakness” (6%), “Retirement” (4%), and “Memory Loss” (2%). Furthermore, when asked what age someone genuinely becomes old, 4% answered 40+, 10% answered 50+, 20% answered 60+, 36% answered 70+, 20% answered 80+, and 8% answered 90 or above.

Participants were worried about becoming dependent on others in old age, with 41.66% saying they were often, or always, worried about it. This preceded death (41.30%), cancers (39.13%), physical weakness (36.73%), memory issues (35.41%), financial issues (31.25%), urinary issues (25.63%), boredom (22.92%), and loneliness (22.45%) (Table 2).

**TABLE 2:** Participant responses to the 17 Likert scale questions (all participants).

	Always *N* (%)	Often *N* (%)	Occasionally *N* (%)	Rarely *N* (%)	Total row *N*
*What do you worry most about aging for yourself?*					
You will be alone or lonely	4 (8.16)	7 (14.29)	18 (36.73)	20 (40.82)	49
Physical weakness	5 (10.2)	13 (26.53)	23 (46.94)	8 (16.33)	49
Memory issues	7 (14.58)	10 (20.83)	19 (39.58)	12 (25.00)	48
Urinary issues	4 (8.16)	9 (18.37)	18 (36.73)	18 (36.73)	49
Financial issues	5 (10.42)	10 (20.83)	11 (22.92)	22 (45.83)	48
Boredom	5 (10.42)	6 (12.5)	9 (18.75)	28 (58.33)	49
Becoming dependent on others	7 (14.58)	13 (27.08)	14 (29.17)	14 (29.17)	48
Cancers	2 (4.35)	16 (34.78)	7 (15.22)	21 (45.65)	46
Death	8 (17.39)	11 (23.91)	7 (15.22)	20 (43.48)	46

*Does the idea of getting old…*					
Frighten you	6 (12.24)	6 (12.24)	18 (36.73)	19 (38.78)	49
Depress you	2 (4.17)	4 (8.33)	13 (27.08)	29 (60.42)	48
Inspire you	9 (18.75)	14 (29.17)	12 (25.00)	13 (27.08)	48
Make you happy to have lived	23 (47.92)	13 (27.08)	8 (16.67)	4 (8.33)	48

*When older people don’t work anymore, they…*					
Enjoy life	7 (14.29)	12 (24.49)	20 (40.82)	10 (20.41)	49
Contribute to society	13 (27.66)	12 (25.53)	16 (34.04)	6 (12.77)	47
Can’t take care of themselves anymore	4 (8.51)	8 (17.02)	24 (51.06)	11 (23.4)	47
Can advise or mentor others	22 (45.83)	18 (37.5)	5 (10.42)	3 (6.25)	48

When asked who they would prefer to live with as they get older, 75.56% said they would prefer to live with their spouse only, 8.89% chose either child, 4.44% chose sons, 4.44% chose friends, 4.44% chose themselves, 2.22% chose an older adult community, and 0% chose daughters. The thought of getting older often or always frightened 24.48% of respondents, depressed 12.5% of respondents, inspired 47.92% of respondents, and made 75% of respondents happy to have lived.

Notably, 60% of participants said they would choose to take care of an older person, such as a relative or friend, while 36% answered maybe and 4% answered no. However, only 31.25% would ever choose to go into a specialty like aging, while 37.50% might and 31.25% would not. When asked what the advantages of a specialty like aging were, 82.22% believed older people are fun to be with, 70.83% believed that older people are medically complex, and 85.11% believed that it is intellectually stimulating.

Participants were asked what the fastest growing age group in the United States is. In correct order, 2.02% answered that the 85–95‐year‐old group was growing the fastest, followed by 75–85‐year‐olds (4.08%), 65–75‐year‐olds (24.49%), 55–65‐year‐olds (38.78%), 35–55‐year‐olds (26.53%), and 15–35‐year‐olds (4.08%).

When older people do not work anymore, 38.7% of participants said older adults enjoy life often or always. Additionally, 53.19% answered that older people can contribute to society often or always when they no longer work. However, 25% answered that older people often or always can no longer take care of themselves, and 83.33% believe that older people can often or always advise or mentor others when they no longer work.

### 3.3. Descriptive Comparison of Survey Responses From Younger and Middle‐Aged/Older Participants

**FIGURE 1:**
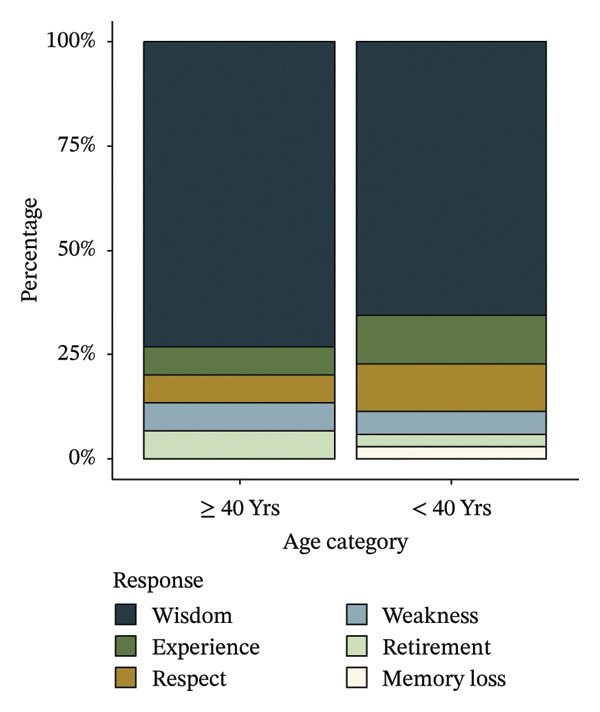
Percentage of participants who chose each term first when asked to rank terms associated with the word “old,” stratified by age group.

Stratifying by age group, when asked to rank terms associated with the word old, participants over 40 answered “Wisdom” (73.3%), “Experience” (6.7%), “Respect” (6.7%), “Retirement” (6.7%), and “Weakness” (6.7%) as the most thought of terms. Those under 40 responded “Wisdom” (65.7%), “Experience” (11.4%), “Respect” (11.4%), “Weakness” (5.7%), “Memory Loss” (2.9%), and “Retirement” (2.9%) (Figure 1).

Furthermore, when asked the age at which a person genuinely becomes old, those over 40 answered 40+ (13.3%), 50+ (13.3%), 70+ (33.3%), 80+ (26.7%), and 90 or above (13.3%). The below 40 group answered 50+ (8.6%), 60+ (28.6%), 70+ (37.1%), 80+ (20.0%), and 90 or above (5.7%).

Notably, 65.6% of respondents under 40 answered they would prefer to live with their spouse only, followed by either child (12.5%), sons (6.3%), friends (6.3%), themselves (6.3%), an older adult community (3.1%), and daughters (0%). Similarly, 100% of respondents above 40 answered that they would prefer to live with their spouse only.

### 3.4. Exploratory Comparison of Survey Responses From Younger and Middle‐Aged/Older Participants

The internal consistency of the 17 Likert scale items was assessed to determine scale reliability. The items demonstrated moderate reliability (ordinal Cronbach’s *α* = 0.77), supporting their use in exploratory inferential comparisons.

Before adjustment of the critical values, a Mann–Whitney *U* test of the responses to the 17 Likert scale items found that participants over 40 were less frightened by death than those below 40, with 91% of respondents in the middle‐aged group answering that they rarely or occasionally worried about death compared with 47.1% of those in the younger group. Additionally, those over 40 were less frightened of getting old, with 92.9% of middle‐aged respondents rarely or occasionally frightened compared to 68.6% of the younger respondents (Figure 2). However, a Benjamini–Hochberg correction of the critical values to control the false discovery rate found these differences to be nonsignificant (Table 3). No comparisons other than those for worriedness about death and fear of getting old were initially promising.

FIGURE 2(a) Percentage of participant responses to “What do you worry about aging for yourself?—Death,” stratified by age group. (b) Percentage of participant responses to “Does the idea of getting old…—Frighten you?” stratified by age group.

**Figure:**
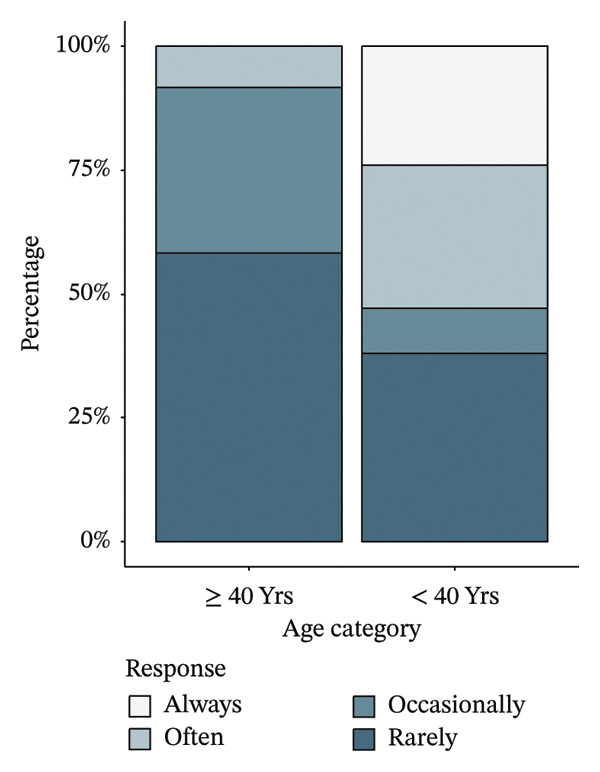
(a)

**Figure:**
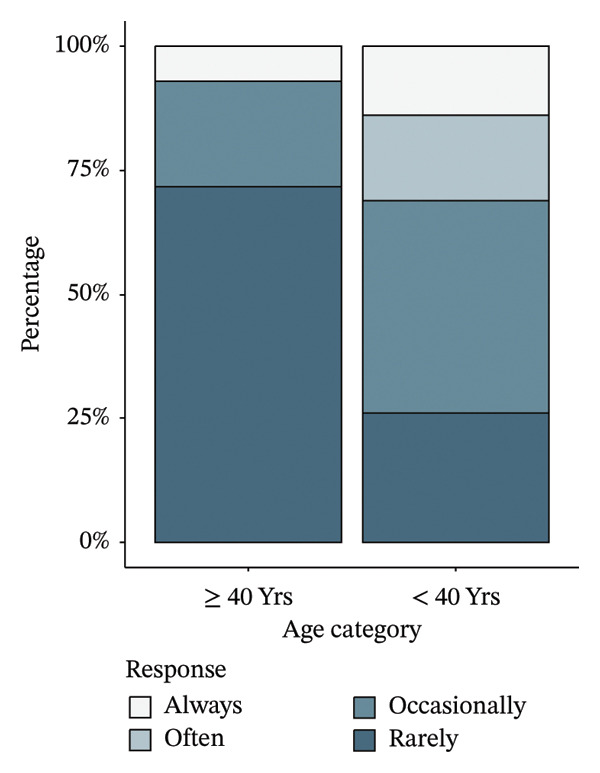
(b)

**TABLE 3:** Results of Mann–Whitney *U* tests performed on responses to each ordinal Likert scale item for younger participants (< 40) and middle‐aged/older participants (≥ 40).

	*p*	Rank (*i*)	Critical value (*i*/*m*) × *Q*
*What do you worry most about aging for yourself?*			
You will be alone or lonely	0.425	11	0.032
Physical weakness	0.602	15	0.044
Memory issues	0.100	6	0.018
Urinary issues	0.081	5	0.015
Financial issues	0.638	16	0.047
Boredom	0.070	4	0.012
Becoming dependent on others	0.289	8	0.024
Cancers	0.509	14	0.041
Death	0.037^∗^	2	0.006

*Does the idea of getting old…*			
Frighten you	0.005^∗^	1	0.003
Depress you	0.298	9	0.026
Inspire you	0.489	12	0.035
Make you happy to have lived	0.817	17	0.050

*When older people don’t work anymore, they…*			
Enjoy life	0.346	10	0.029
Contribute to society	0.270	7	0.021
Can’t take care of themselves anymore	0.495	13	0.038
Can advise or mentor others	0.057	3	0.009

*Note: p*‐values are ranked in ascending order. Critical values were adjusted using the Benjamini–Hochberg procedure with a false discovery rate of *Q* = 0.05 and *m* = 17.

^∗^Asterisk indicates that adjustment of critical value changed the hypothesis decision.

## 4. Discussion

This study examined the perceptions of Hispanic adults about aging. The results of this study suggest that Hispanic adults have a generally positive view of aging. Across younger and older groups, the majority of respondents associated wisdom with old age and believed that older adults can advise or mentor others. These findings are consistent with other research, emphasizing the importance of respecting elders within the Hispanic community and highlighting potential culturally rooted values that serve as protective factors in aging [39]. Additionally, most respondents were happy to have lived and inspired by the thought of growing older. Previous research also shows Hispanics to be particularly optimistic, with one study showing that poor Hispanics and non‐Hispanic blacks were significantly more optimistic than poor non‐Hispanic whites, even under conditions of socioeconomic disadvantage [40].

Despite these positive views, concerns about aging were still prevalent. Particularly, respondents expressed concerns about dependence on others in old age and fear of death. In another study, cognitively impaired older Hispanic adults marked their fear of losing independence and becoming a burden to their children [41]. While this study could not definitively determine differences in these concerns between younger and older adults due to sample limitations, a more in‐depth exploration of how perceptions on aging may vary across the lifespan within the Hispanic community is warranted. Noting these concerns will be critical for healthcare providers when developing care options that promote well‐being for the Hispanic older adult population.

Previous studies have shown a strong tendency for Hispanic older adults to live in complex households, including their spouses, children, and other relatives [42, 43]. Similarly to Asian and Black Americans, about a quarter of Hispanic Americans in the United States reported living in multigenerational households in 2021 [44]. This is double the rate of non‐Hispanic White Americans. Interestingly, in this study, the majority of participants reported that they would prefer to live with their spouse only when they are older. This may reflect generational differences in attitudes toward living arrangements or a desire for financial independence, but these findings should be interpreted cautiously due to the limited sample. All participants in the middle‐aged/older group indicated a preference to live with their spouse, which may be a reflection of their life stage (mostly 40s and 50s) and perhaps better‐preserved physical functional status. One study examining the effects of social exchange variables on the expectation of filial piety found that younger age was significantly associated with a lower expectation of filial piety [45]. Further research involving more participants in their 60s and older, as well as measures of household structure, is needed to clarify these dynamics.

Compared to other populations, older Hispanic adults are less likely to use nursing homes or professional caregiving, instead relying on family for caregiving services [46–48]. However, some studies have indicated that this discrepancy could be a result of systemic and economic barriers rather than cultural. Like Hispanics, non‐Hispanic blacks report fewer socioeconomic resources because of discrimination processes and report lower nursing home admissions [49]. In this study, more than half of adults surveyed answered that they would serve as a caregiver to a family member. This could indicate that filial responsibility still plays a significant role in the culture of younger Hispanic adults; however, there is a lack of research to suggest that this differs across ethnic lines [50]. These results, consistent with other studies, suggest that the concepts of familismo, personalismo, and respeto still significantly influence the decisions of Hispanic adults when it comes to caregiving and healthcare [51, 52]. Particularly, these concepts may simultaneously promote strong intergenerational support while discouraging the utilization of formal caregiving. Further research could explore how these values intersect with socioeconomic status.

Research suggests that financial anxiety can negatively impact mental well‐being and healthcare utilization [53, 54]. One study examining financial worry among adults in the United States found that Hispanic adults had greater financial stress when compared to non‐Hispanic blacks and non‐Hispanic whites [55]. Additionally, Hispanic adults are less likely to seek financial planning advice when compared to other ethnic groups [56]. As of 2022, 18% of Hispanics or Latinos in the United States were uninsured, higher than any other racial or ethnic group and more than double the rate of non‐Hispanic whites [57, 58]. Being uninsured is significantly associated with lower healthcare utilization and poorer health outcomes in cancer patients [59]. In this survey, more than half of participants responded that they were at least occasionally concerned about financial issues in older age. This suggests the need for the creation of and improved access to culturally competent financial planning and insurance education services that are accessible and family‐centered to mitigate economic barriers to healthcare among Hispanic adults [60, 61].

Furthermore, the concern of respondents about becoming dependent on others at an older age highlights the importance of tailored educational programs to provide the Hispanic population with opportunities to learn about caregiver resources and healthcare in older age [62]. Previous studies have shown that targeted educational content can significantly improve the willingness of Hispanic caregivers to accept help [62]. Such content could address how to recognize when formal caregiving services are needed and how to access culturally sensitive care [63]. The findings of this study emphasize the importance of culturally responsive care practices in which formal caregivers treat patients with respect and allow patients to maintain autonomy where possible. Additionally, it is important for providers to be culturally sensitive and recognize that family values, systemic barriers, and linguistic factors can play an important role when it comes to making healthcare decisions for older Hispanic adults [64].

## 5. Strengths and Limitations

A key strength of this study is that it pulls from a diverse socioeconomic background, with representation of different educational and socioeconomic groups. Further, there is a dearth of studies examining the opinions of the younger Hispanic adult population on aging, making this study unique.

The study suffers from the limitation of small sample size, making it difficult to determine whether the results are truly representative of the larger Hispanic community. Particularly, the effect sizes when comparing responses from younger and middle‐aged/older participants may have been more effectively captured by a larger study. The use of Mann–Whitney *U* tests, especially with corrections for multiple comparisons, results in this study being underpowered. Additionally, the use of a convenience sample design has the potential to introduce sampling bias due to the selection of participants who were available to take the survey rather than drawing a random sample. Furthermore, we recognize that Hispanic Americans are not a homogenous group, and this survey, which consisted of white and largely Mexican American and Central American participants who speak English as a first or second language, may not be generalizable to the larger Hispanic community in South Central United States.

## 6. Conclusion

In conclusion, this study serves as a novel glimpse into perceptions that the Hispanic community in the South Central United States holds toward aging. These findings highlight the positive perceptions that Hispanic adults hold toward aging and identify age‐related anxieties such as fear of death and dependence on others that may be addressed through culturally competent programs and healthcare. As we aim to promote healthy aging, we need to explore the viewpoints of Hispanic people living in different regions of the country and develop educational programs that will help sustain and improve the overall health of the Hispanic community. In the future, larger studies should be conducted that include further information on immigration, education and employment background, access to healthcare, language use, and acculturation. These details will allow for a more nuanced and representative analysis of Hispanic perceptions toward aging that may inform future programs and research.

## Funding

This work was supported by the Lyon Aging Research Program at the Reynolds Institute on Aging, Little Rock, AR.

## Conflicts of Interest

The authors declare no conflicts of interest.

## Data Availability

Data will be shared upon request.
